# Paying attention to attention in depression

**DOI:** 10.1038/s41398-019-0616-1

**Published:** 2019-11-07

**Authors:** Arielle S. Keller, John E. Leikauf, Bailey Holt-Gosselin, Brooke R. Staveland, Leanne M. Williams

**Affiliations:** 1 Graduate Program in Neurosciences, Stanford University, Stanford, CA USA; 2 Department of Psychiatry and Behavioral Sciences, Stanford University, Stanford, CA USA

**Keywords:** Depression, Human behaviour

## Abstract

Attention is the gate through which sensory information enters our conscious experiences. Oftentimes, patients with major depressive disorder (MDD) complain of concentration difficulties that negatively impact their day-to-day function, and these attention problems are not alleviated by current first-line treatments. In spite of attention’s influence on many aspects of cognitive and emotional functioning, and the inclusion of concentration difficulties in the diagnostic criteria for MDD, the focus of depression as a disease is typically on mood features, with attentional features considered less of an imperative for investigation. Here, we summarize the breadth and depth of findings from the cognitive neurosciences regarding the neural mechanisms supporting goal-directed attention in order to better understand how these might go awry in depression. First, we characterize behavioral impairments in selective, sustained, and divided attention in depressed individuals. We then discuss interactions between goal-directed attention and other aspects of cognition (cognitive control, perception, and decision-making) and emotional functioning (negative biases, internally-focused attention, and interactions of mood and attention). We then review evidence for neurobiological mechanisms supporting attention, including the organization of large-scale neural networks and electrophysiological synchrony. Finally, we discuss the failure of current first-line treatments to alleviate attention impairments in MDD and review evidence for more targeted pharmacological, brain stimulation, and behavioral interventions. By synthesizing findings across disciplines and delineating avenues for future research, we aim to provide a clearer outline of how attention impairments may arise in the context of MDD and how, mechanistically, they may negatively impact daily functioning across various domains.

## Why characterize attention in depression?

Cognitive dysfunction is included as a diagnostic criterion for major depressive disorder (MDD), described as “Diminished ability to think or concentrate”^1^. These cognitive problems may include impairments in executive functions, learning and memory, processing speed, as well as in concentration and attention^2^, and these are associated with a disproportionately poor prognosis in psychosocial and occupational domains. Attention impairments in particular are known to negatively impact daily function^3,4^ and are associated with poorer clinical outcome^5^. Although cognitive dysfunction is a hallmark of MDD contributing to disability, it is not well understood, especially in comparison with the mood features of MDD.

Our focus on attention as a specific cognitive domain aligns with the matrix of cognitive neurobiological constructs within the Research Domain Criteria (RDoC) framework, intended to advance a precision medicine approach to psychiatry informed by neurobiology^6^. The cognitive systems domain of RDoC comprises a construct of attention as well as constructs of perception, declarative memory, language, cognitive control, and working memory^7^. RDoC also includes both the negative and positive valence systems and associated constructs of emotional function and mood. Thus, the RDoC matrix provides a framework from which to hone in on attentional impairments in MDD and to consider how attention may influence other domains of the cognitive and emotional systems. Although it is often assumed that group-level deficits in a variety of cognitive tasks^8^ imply that depressed individuals experience a “general” cognitive deficit, there is not yet sufficient evidence to suggest that an individual with deficits on one cognitive task will necessarily exhibit deficits on other tasks. Therefore, we focus on parsing impairments in specific sub-domains of goal-directed attention, and outline directions for future research to parse individual cognitive impairments with further granularity.

In the following review, we hone in on dysfunction in attention, which may encompass several sub-domains of patient experiences, from increased distractibility (selective attention impairment) to an inability to sustain focus (sustained attention impairment) or an inability to simultaneously monitor multiple channels of information (divided attention impairment). We seek to characterize these specific types of attention impairments in MDD and their neurobiological correlates, informed by current insights from cognitive neuroscience and the current state of knowledge about such impairments in MDD and the mechanisms by which they might develop and impact other areas of cognitive and emotional function. Given that attention and other cognitive dysfunctions in MDD are associated with poor outcomes following treatment with current standard-of-care interventions, we also review potential alternative treatments to address attention impairment with greater precision and consider the case for longitudinal studies.

### Domains of goal-directed attention and impairments in MDD

We operationalize top–down attention, defined within cognitive neuroscience as “guidance of attention based on prior knowledge, willful plans, and current goals”^9^. Here, we review comparisons of behavioral performance between MDD patients and healthy controls on three sub-domains of top–down attention (selective, sustained, and divided attention) defined below. In particular, we focus exclusively on studies using *neutral* stimuli (e.g. arrowheads) to probe top–down attention capabilities, to complement the extensive literature regarding emotionally guided attention and negative attentional biases (discussed separately in section “Attention and the negative valence system”; for discussion of internally vs. externally guided attention see Supplementary Appendix). Given that MDD is a highly heterogeneous disorder^10^, we expect that attention impairments may not always be present for all depressed individuals, supported by the observation that not all depressed individuals endorse attention problems as a core symptom. Thus, we take a relatively conservative approach to uncovering attention impairments by reviewing findings at the group level.

### Selective attention impairment

Selective attention is the ability to attend to important (task-relevant) information while ignoring distracting (task-irrelevant) information. Over a century ago, this dual process of simultaneously attending and ignoring was proposed to be essential for parsing the overload of input to our perceptual systems^11^ and a breakdown of these processes would result in a more distracted, overwhelmed, and confused state. Within the cognitive neuroscience literature, selective attention is discussed a multifaceted construct in of itself. One important distinction is whether task-relevance is defined by stimulus features or by its spatial location. Feature-based selective attention occurs when one attends to a particular aspect of the available information (e.g., color or shape), while ignoring other, irrelevant features. Spatial selective attention occurs when one must attend to information in a particular area of space (e.g., left or right visual field) while ignoring information in irrelevant locations. Whether these two forms of selective attention rely on dissociable neural mechanisms has been a controversial topic among neuroscientists studying visual attention. Some researchers have argued that feature-based and spatial selective attention involve activity modulation in similar regions^12^ and result in additive modulation when used together^13^. Others have demonstrated slight differences in the particular sub-regions of frontal and parietal cortices modulated by these subtypes of selective attention^14,15^. Clinical studies aimed at understanding the distinct and overlapping behavioral and neural phenotypes of feature-based and spatial selective attention impairments may provide further insight into this key question in cognitive neuroscience in addition to clarifying the specific impairments observed in depression.

Many investigations of color-word reaction times have revealed that unmedicated depressed individuals (including children, adolescents, and adults) perform worse than healthy controls when required to attend to a task-relevant feature (e.g., color) while simultaneously ignoring a distracting feature (e.g., semantic meaning)^16–19^, including in an international sample of *n* = 1008 MDD participants without co-morbid ADHD^20^. Importantly, these studies measured response times in either the word- or color-naming condition independently to capture feature-based selective attention abilities, rather than measuring inhibitory control using the classic measure of “interference” calculated by subtracting reaction times in the color-naming condition from those in the word-naming condition. More often than not, studies investigating spatial selective attention by using the Flanker task (identifying the direction of an arrow flanked by distracting arrows) do not observe a significant difference in performance between unmedicated MDD patients and healthy controls^21,22^. This might suggest that selective attention impairments in MDD are specific to feature-based selective attention while spatial selective attention remains intact which could imply that (1) not all of the underlying neural mechanisms are shared between feature-based and spatial selective attention and (2) cognitive impairments in the context of depression may not be as homogeneous as previously thought. However, it is important to note that the Flanker task used to assess spatial attention tends to be much less challenging than color- and word-naming tasks, with performance close to ceiling. Future investigations could use equally difficult versions of feature- and spatial-selective attention tasks to determine whether this deficit is truly specific to the feature-based case.

### Sustained attention impairment

Sustained attention refers to the ability to continuously attend to, or monitor, task-relevant information, usually assessed in the relative absence of distractions (e.g. in a quiet testing room). When sustained attention is impaired, one might report an inability to maintain focus at work or school. To assess the degree to which someone is sustaining attention on a set of stimuli, researchers most commonly present intermittent “oddball” stimuli (e.g., the Continuous Performance Task) and measure the speed of detection. It is assumed that subjects who successfully engage in sustained attention will have faster hits and fewer misses when these oddball stimuli appear. Studies comparing oddball task performance by unmedicated MDD patients versus healthy controls often find that MDD patients respond more slowly than controls^23–25^, suggesting that even in the absence of overt distraction attentional focus is impaired. However, other studies in both adolescents and adults fail to find a significant difference in oddball reaction times between MDD patients and healthy controls^26,27^. Future meta-analyses may reveal whether these differences reflect true inter-subject heterogeneity or measurement variability. Recent work investigating the impact of rumination on cognition in depressed individuals has proposed that rumination may underlie observed deficits in sustained attention by competing for attentional resources^28^. Similarly, excessive worrying in the context of depression or anxiety may also sap attentional resources. In our own data, we do not observe a significant correlation between *selective attention* and worrying^20^, but future work may reveal whether excessive worrying and/or rumination contribute to inter/intra-individual differences in *sustained attention* deficits.

### Divided attention impairment

Divided attention refers collectively to the functions of multi-tasking and of simultaneously attending to multiple sources of task-relevant information, which is often critical for efficient daily functioning. One of the first controlled studies of divided attention used a dichotic listening task to demonstrate the inherent difficulty of parsing two different messages delivered to each ear simultaneously^29^. Although the challenges of divided attention have since been documented extensively^30^, it was not until lesion studies in the 1990s that the neural basis of these difficulties began to be understood. These early neuroscience studies demonstrated the importance of the prefrontal cortex in attending to multiple sensory modalities simultaneously^31^, performing multiple tasks simultaneously^32^, and switching attention among tasks^33^. In the context of MDD, studies of unmedicated adults have revealed impairments in performing two tasks simultaneously^34^, and simultaneously attending both auditory and visual stimuli to detect targets^35^ compared with healthy controls. Better divided attention appears to predict treatment efficacy independently of baseline depression severity^36^ while poorer divided attention is associated with delayed response and increased risk of relapse^5^ as well as higher suicidality^37^.

## Attention and cognition

The ability to allocate one’s attention volitionally in selective, sustained, or divided attention contexts is critical for a variety of cognitive tasks. Thus, impairments of goal-directed attention may have downstream effects on other functions, such as cognitive control, perception, and decision-making. Here, we discuss the relationship between top–down attention and other cognitive domains to shed light on their interactions in depression.

### Cognitive control

Much work has been done to advance our understanding of how various cognitive control functions may change in the context of MDD (for review^38,39^). According to the RDoC working group^40^, the broad construct of “cognitive control” encompasses sub-functions, such as the selection and updating of goal representations, response selection and suppression, and performance monitoring. These cognitive control functions are also known in the neurocognitive literature as aspects of executive function, and many of these sub-functions also involve aspects of top–down attention (e.g., re-allocation of attention toward goal-relevant information upon encountering feedback of a performance error). The RDoC working group states that “cognitive control most often requires attentional processes, and thus cognitive control tasks also test attention” and similarly, Chun et al.^41^ state that “To the extent that there are limitations in the number of alternatives that can be considered at any given time— and even broader set of responses and choices that can be made to these alternatives—cognitive control is intrinsically attentional.” Moreover, cognitive control processes and top–down attention often share overlapping neural circuitry (see the section “Putative mechanisms of attention impairment”).

This close relationship between attention and cognitive control presents a challenge for characterizing specific deficits and their biological substrates in psychiatric illness. To thoroughly examine top–down attention as our construct of interest, we focus exclusively on studies which measure participants’ ability to allocate attention volitionally in selective, sustained, or divided attention contexts (as operationalized in the section “Domains of goal-directed attention”), regardless of whether these functions are referred to in the literature as top–down attention, cognitive control, or executive functioning. In particular, when examining studies of feature-based selective attention using the Stroop task, we limit our review to studies using raw reaction time measurements with either color or word stimuli independently (in accordance with the definition of feature-based selective attention as attending relevant information while ignoring distraction) and exclude studies which use interference scores calculated by subtracting reaction times in two conditions (conceptualized as a measure of cognitive control). Further discussion of the behavioral and neurobiological distinctions between top–down attention and cognitive control can be found elsewhere^42^.

### Perception

Once considered a bottleneck limiting sensory processing, attention is now appreciated as a critical gating mechanism for sensory perception, helping to form our fluid, organized, conscious experience from the abundant information bombarding our sensory systems. More recently, cognitive psychological studies have characterized the particular ways, in which attention can influence perception, from low-level visual features to high-level perceptual judgments (for review see ref. ^43^). For example, a large body of research has demonstrated that both automatic attention and voluntary attention to particular visual stimuli increases perception of both contrast and color saturation^44,45^. Higher-level perceptual features such as the attractiveness^46^ or the intensity of emotional expression in a face^47^ are also altered by attention. The finding that attention substantially alters the overall appearance of sensory information suggests that attention impairments may impact perception. These findings are in accordance with research at the sensory level, where dramatic differences in retinal contrast gain have been observed between depressed individuals and healthy controls^48^. Barbot and Carrasco^49^ recently showed that emotion and trait anxiety moderate the effect of attention on perceived contrast, motivating future studies to provide more detailed characterizations of interactions between depressive symptoms and the effect of attention impairments on perception.

### Decision-making

In addition to the profound influence of attention on perception, attention also appears to play a critical role in decision-making. A plethora of research has revealed that we are more likely to choose options that we have attended to for longer regardless of the subjective value of those options (for review see ref. ^50^). Indeed, gaze bias appears to both reflect and influence preferences^51^, and reward-learning in turn affects the allocation of attention with strong biases toward previously rewarded locations^52^. Recent advances using computational modeling of behavior have shown that selective attention is a requirement for effective multidimensional reinforcement learning^53^, and that attention influences the choices we make as well as our learning of reward associations over time^54^. Efforts in computational psychiatry have sought to characterize changes in decision-making processes in the context of depression and have revealed dysfunction in the processes underlying model-based decision-making (for review see ref. ^55^). Future work may further elucidate the reciprocal interplay of impaired attention and decision-making in depression by leveraging knowledge across disciplines.

## Attention and the negative valence system

### Negative attentional biases

Negative attentional biases offer one way to consider how attention (within the cognitive system of RDoC) may interact with processes within the Negative Valence system. A large body of research, largely utilizing the Dot Probe^56^ or Emotional Stroop Task^57^ has shown that depressed patients tend to spend more time attending to negative information, such as sad faces than neutral or positive information (for review see refs. ^58,59^). Generally speaking, depressed patients’ attention tends to linger longer on negative information such as sad faces than healthy controls do, which suggests a negative bias in the way that depressed individuals sample information from their environments. However, given the substantial evidence (reviewed above) that depressed individuals suffer from attention dysfunction in *neutral* contexts compared with healthy controls, characterizations exclusively focusing on negative biases in attention do not provide a full picture. It is plausible that these negative attentional biases are exacerbated by general impairment of top–down attention allocation (and re-allocation toward goal-relevant information when something has inadvertently caught one’s attention). In other words, depressed individuals may have their attention initially captured by salient negative information^60^, but the *lingering* of attention on this negative information may be due to an inability to *re-orient* attention away from distracting negative information toward goal-relevant information. Given that persistent low mood may be perpetuated^61^ by negative attentional biases in MDD breaking this cycle of attention-related biases has the potential to yield substantial improvement in wide-ranging symptomatology and overall quality of life.

### Mood and attention

Conversely, mood states may also influence the ways that we allocate attention, whether consciously or unconsciously. Theories about how attention changes in different mood states have been wide-ranging (for review see ref. ^62^). Some suggest that positive mood leads to decreased attention and cognitive effort. For example, the “mood-as-input” theory^63^ postulates that positive mood makes tasks more enjoyable and could render subjects more easily satisfied with lower performance. Other theories suggest that positive mood actually *improves* attention, while low mood is associated with worse attention. For example, the “broaden-and-build” theory^64^ suggests that positive mood enhances cognition by making it broader, emphasizing increased creativity^65^, and flexible thinking^66^, as well as broadened attentional scope^67^. This model is in accordance with early theories of negative arousal narrowing attentional focus^68^, such as heightened attention to a threatening weapon and diminished attention to other details^69^. Brand et al.^70^ showed that mood induction (e.g., euphoric or distressing film fragments) in healthy adults could influence selective attention abilities on a nonemotional Stroop task, in accordance with the findings reviewed above of diminished selective attention performance in MDD patients. These findings suggest that, as theorized by the “affect-as-information” framework^71^, emotional/mood states can influence attention and overall cognitive styles, thus low mood observed in depression may contribute to attentional impairments and attentional impairments may in turn perpetuate low mood.

## Putative mechanisms of attention impairment

### Neural circuits

Understanding the biological correlates of attention allocation and the ways in which they may become disrupted is a critical first step toward developing more targeted treatments. Neuroimaging studies using a variety of attention tasks^14^ have identified a common network, often referred to as the fronto-parietal attention/control network^72–74^. This network, which includes areas such as the frontal eye fields, intra-parietal sulcus, medial prefrontal cortex (mPFC), and superior parietal lobule, appears to be critical for the control of goal-directed attention^75^ with increasing involvement for more difficult tasks^76^. In our own work, we have directly linked hypoconnectivity within this fronto-parietal attention network to poorer goal-directed attention in MDD^20^.

More recently, studies using unbiased data-driven approaches (complementing hypothesis-driven studies) have independently confirmed the importance of the fronto-parietal attention network for coordinating cognition and its dysfunction in MDD using graph theoretical metrics applied to analyses of network activity. Specifically, Bassett et al.^77^ showed that flexibility of nodes within this network predicts subjects’ learning of a simple motor task with visual cues at a future time point, and Gu et al.^78^ demonstrated that the fronto-parietal attention network has high modal controllability, meaning that it is capable of affecting a wider range of possible neural states, including harder-to-reach states, than other large-scale networks (in line with the overarching finding that regions with a large number of long distance connections tend to be optimal controllers^79^. These findings begin to provide a mechanistic account for how attention may influence a variety of important functions. Studies of topological organization in depressed individuals using anatomical^80^, resting-state^81^, and task-based functional connectivity^82^ have found disruption to this same fronto-parietal network. This developing literature suggests that changes to fronto-parietal network function in the context of depression may represent an under-investigated target for the development of symptom-specific treatment.

It is important to acknowledge that even with significant advances in neurobiological studies of attention, our understanding of precisely which neural systems support which sub-functions of attention remains murky. Recent data-driven approaches to unpacking subnetworks within the fronto-parietal attention system have yielded varying results, often accompanied by even more variable naming schemes^83,84^. Many attempts have been made to distinguish these subnetworks based on independent sub-functions of attention, such as a “dorsal attention network” involved in top–down allocation and a “ventral attention network” involved in bottom-up re-orienting, or a “central executive network” involved in goal-oriented attention and a “cingulo-opercular network” involved in salience-driven attention. These distinctions, however, remain unclear and inconsistent, with more recent studies providing counter-evidence to this dogma of distinct attention networks for bottom-up and top–down processing^85^. Even with the increasing number of studies using data-driven approaches to disentangle attention-related subnetworks, most of these approaches assume network independence and orthogonality rather than addressing the potential for spatial overlap in network architecture. Studies that attempt to capture the complexity of the attention system have taken various approaches such as accounting for time-varying changes in network arrangement by task state^86^ or in fast alternating rhythms within a task^87^, addressing differences in network configuration during attention to distinct sensory modalities^88,89^. In order to develop clinically applicable biomarkers of this dynamic and complex attentional system, we will need to take into account variability on all fronts, from individual differences to task-related differences to temporal reconfigurations.

### Oscillatory synchrony

With abundant sensory information all around, the neurobiological mechanisms supporting ignoring of irrelevant information may be just as important as those supporting attentional orienting and focus. Electroencephalography (EEG) studies have shown that cortical oscillations in the alpha band (8–14 Hz), previously considered an “idling” rhythm increasing in power at rest^90^, has more recently been linked to ignoring of task-irrelevant information (for review see ref. ^91^). Increased power in the alpha band has been linked to suppression of sensory signals^92,93^, and appears to play a causal role in suppressing the intrusion of distracting information when applied via transcranial magnetic stimulation during a visual target detection task^94^. Fronto-central theta oscillations (4–7 Hz) appear to play a more executive role in orchestrating the guidance of top–down attention toward task-relevant information and switching the focus of attention among various stimuli (for review see ref. ^95^). In support of this theory, fronto-central theta power has been associated with novelty detection and goal-directed responses^96^, as well as divided attention between conflicting visual and auditory signals^97^. Together, alpha and theta oscillations appear to support normal attention function and may be a potential substrate of attention dysfunction in disorders such as depression.

Combining information across neuroimaging and electrophysiological studies, it has been shown that the strength of theta synchrony within the fronto-parietal attention network can predict goal-directed attention behavior^98^ and thus may be a potential candidate for investigating the neural substrates of attention dysfunction in depression. Recent evidence has shown that fronto-parietal theta appears to set a clocking rhythm for oscillations at other frequencies such as alpha during rhythmic alternations between attention and ignoring—a phenomenon referred to as “theta-dependent” perceptual sampling^87^. Further investigations of these spatio-temporal dynamics may reveal important neurobiological substrates for the development of targeted treatments for attention impairments in depression.

## Improving attention

Treatment trials in depression have largely focused on clinical measures of response rather than on behavioral measures of attention and very few report item-level data, making it challenging to parse out specific changes in attention. Here, we review potential pharmacological, brain stimulation, and behavioral interventions and their effects on sub-domains of goal-directed attention (selective, sustained, and divided) in the currently available literature.

### Pharmacological interventions

Unfortunately, selective serotonin reuptake inhibitors (SSRIs), currently the first-line class of pharmacologic treatment for depression, have generally not been shown to improve attention. Many studies, albeit largely observational or with a mix of medications used, show no change in sustained attention^25,99^, selective attention^100^, or divided attention^5,101^, despite improvements in mood symptoms. A recent systematic review of healthy individuals even found evidence for worsening of divided and sustained attention with SSRI treatment^102^. Vortioxetine, a relatively newer serotonin reuptake inhibitor (SRI) with mixed agonist/antagonist/partial-agonist effects, is a notable exception that has demonstrated benefits for sustained and selective attention as well as depressive symptoms in adults with MDD^103,104^.

Catecholaminergic agents, some of which are approved for depression but many of which have been approved for other disorders such as attention-deficit/hyperactivity disorder (ADHD), have a much stronger evidence base for improving attention and other aspects of cognition. We briefly review findings regarding the effects of some of the more commonly used of these medications on attention. While the potential side effects, including abuse, must be managed carefully, future studies should investigate the ability of agents such as psychostimulants or modafinil to improve attention in depression as a clinical target with important functional implications in its own right that are not well addressed by SSRIs. Norepinephrine (NE) and dopamine (DA) are believed to modulate attention and other cognitive capabilities^105^, generally with dose–response following classic inverted-U shaped curves. As with MDD, persons with ADHD frequently have deficits in goal-directed attention. Psychostimulants, which increase levels of both NE and DA in the striatum and cortex, remain the first-line treatment for ADHD and have also been used as an adjunctive therapy in MDD despite limited support from high quality, randomized, controlled trials that examined mood-related symptom improvement alone^106^.

There is strong evidence that dopaminergic agents can improve sustained attention, and possibly selective and divided attention. This weight of evidence toward sustained attention may in part be an artifact of historical focus on sustained attention as the primary cognitive domain affected in persons with ADHD. Both methylphenidate^107,108^ and amphetamine^109,110^ have been shown consistently to improve sustained attention in both healthy adults and youth and adults with ADHD. Similarly, methylphenidate^111,112^ and amphetamine^113^ have been demonstrated to improve selective attention, while methylphenidate has been shown to improve divided attention^111^. Bupropion, a NE and DA reuptake inhibitor approved as an antidepressant has likewise been shown to improve sustained attention in youth with ADHD^114^ and healthy adults^115^. Modafinil, thought primarily to act as a weak dopamine reuptake inhibitor, has been shown in several randomized trials to be effective for symptoms of MDD as adjunctive treatment (reviewed in ref. ^106^). It can improve both sustained and selective attention in healthy subjects^116,117^, enhance sustained attention in ADHD patients^118^, and increase feature-based selective attention and clinical symptoms in depressed patients^119^. Finally, the D3 agonist pramipexole has recently been shown to improve sustained attention, particularly in baseline low performers^120^.

Selective noradrenergic agonists, including serotonin and norepinephrine reuptake inhibitors (SNRIs), atomoxetine, and the alpha2 agonists have been shown to improve selective^121^ and sustained attention in some studies^103,122^, but not all^123,124^. Some studies have found improvements in selective attention with venlafaxine^122^ and with duloxetine^125^, but the results have been mixed^99,100^. Guanfacine, a direct alpha2a agonist, has been shown to improve feature-based selective attention in at least one study of 17 adults with ADHD^126^. Noradrenaline is a major component of the inverted-U shaped physiologic response to stress, and alpha2a adrenergic receptors in the prefrontal cortex^127^ mediate many aspects of cognitive control^128^. Differences in noradrenergically mediated stress responses, as well as individual differences in response to acute and chronic stress may in part explain why findings are more mixed regarding attention than for dopaminergic agonists in these varied populations. With the importance of chronic stress in depression (section “Stress reactivity and attention”) in mind, future studies might investigate catecholaminergic agents as adjunctive treatment in depression-considering stress and baseline cognitive performance as potential moderator effects–and focus on cognitive outcomes independently of clinical rating scales.

### Brain stimulation

Another possibility for targeted intervention is utilizing noninvasive brain stimulation, including transcranial magnetic stimulation (TMS) and transcranial direct-current stimulation (tDCS). Optimal targets and protocols remain an active area of research, but these interventions are appealing for their potential to target specific anatomic regions and thereby alter network functionality more precisely than chemical neuromodulators^129^. The most common target in MDD has been left dorsolateral prefrontal cortex (DLPFC), which is generally considered to be a node in the cognitive control network, albeit one that interfaces with the fronto-parietal attention network^130^. TMS and tDCS have been evidenced to improve both selective attention via targeting the DLPFC in healthy controls^131^ and sustained attention in depressed adults^132^. Multiple studies have shown successful sustained attentional enhancements in depressed samples via TMS^133^, rTMS^134^, and tDCS^135^.

However, a recent systematic review found it difficult to draw firm conclusions regarding effects of brain stimulation on attention in depression with several studies not finding effects on Stroop tasks or sustained attention^136^. This finding is in accordance with prior systematic reviews of DLPFC stimulation effects on cognition across psychiatric disorders which found no improvement in attention domains (including selective, sustained, and divided) and only found significant improvements in working memory^137^ and verbal memory^138^. Given that the vast majority of studies investigating cognition in mental illness utilize the DLPFC as a target, it is perhaps unsurprising that functions more closely associated with the DLPFC such as working memory have been observed more readily. It remains unknown whether stimulation of more precise targets in the fronto-parietal network would yield more specific changes in attention function.

### Behavioral interventions

Varied attempts have also been made to improve attention via behavioral interventions, though often outside the context of mental illness. An increasing body of data indicate that physical exercise has preventative and therapeutic effects for mood-related symptoms of depression^139^. Physical exercise has also been demonstrated to improve selective attention behaviorally in both healthy controls^140^ as well as young adult^141^ and older adult MDD patients^142^, though these effects appeared to be short-lived and recent reviews find mixed results^143,144^. Mindfulness meditation (MM) involves rehearsing the skill of selectively attending to one sensation (e.g., breath) while ignoring distracting thoughts^145^, and has been shown to improve selective attention measured independently^146^ in addition to improving mood-related symptoms of MDD^147,148^ and increasing alpha oscillations^149^. A systematic review analyzing 23 studies indicated improvements in selective attention through MM in various nonclinical and clinical samples^146^. More recent studies using neurofeedback-assisted technology-supported mindfulness training (N-tsMT) have revealed selective attention improvement and enhancement in well-being in healthy individuals^150^. Additional research on MM (in person and virtually) in MDD patients with attention impairments in particular is warranted.

Computerized cognitive training apps, which involve cognitive exercises or immersive video games, have also been studied as potential treatments for attentional and mood-related symptoms in MDD^151^. Anguera et al.^152^ found that older adults with MDD exhibited improved sustained attention and mood-related symptoms after four weeks of a mobile cognitive intervention app, and a meta-analysis found that computerized training improved mood symptoms and attention^153^. Notably, the benefits of computerized cognitive training may generalize beyond the specific cognitive tasks practiced, such as improving untrained measures of attention, reducing negativity bias, and enhancing daily functioning, suggesting far transfer effects^152,153^. Additional work will be needed to fully assess far transfer of these various interventions, measuring generalizability of behavioral performance on a wider variety of tasks. Furthermore, these apps have not always outperformed control interventions and tend to have high drop-out rates that are worse with more severe depression^154^. Nonetheless, given that computerized treatments are generally inexpensive, noninvasive, and can be tailored to the individual, these interventions warrant further research.

## Future directions

### Stress reactivity and attention

One theory is that pathology associated with MDD (e.g., neuromodulatory changes, stress-related pathology, etc.) leads to attention impairments as a downstream effect. Here, we describe one potential theoretical model for how stress hyperreactivity associated with recurrent depression might interfere with the fronto-parietal attention network. The canonical stress pathway, involving the hypothalamus–pituitary–adrenal axis, exhibits dysregulation in depression (for review ref. ^155^) associated with increased cortisol^156^ and subsequent neuronal atrophy in the hippocampus^157^ and mPFC^158^. Given the importance of hippocampal-mPFC communication via theta rhythms for a variety of cognitive functions involving top–down attention^95,159,160^, it is possible that stress hyperreactivity could underlie attention impairments by disrupting this mechanism^161^.

Moreover, the fronto-parietal attention network (which also synchronizes in the theta band^98^); includes areas of the mPFC. It is theoretically plausible that these systems work together in healthy adults to translate task/goal representations into attentional control over sensory processing by means of theta synchronization, and that disruption to the mPFC by stress hyperreactivity disrupts this pathway. This model would predict that patients experiencing stress hyperreactivity would also be more likely to have impaired attention. The mPFC is not only a key node of the fronto-parietal attention network; it is also a key hub for emotional processing. Researchers have therefore suggested that the mPFC may act as a cognitive/emotional integration site^162^—a plausible locus for the influence of emotion on guiding attention and vice versa. Given that the mPFC is known to undergo neurodegeneration in the context of stress hyper-reactivity in depression^158^, it may be that as this critical hub region deteriorates both cognitive and emotional symptoms arise together.

### The case for longitudinal studies

Future studies may address whether attention problems are a cause or consequence of mood-related symptoms of depression by characterizing longitudinal trajectories. One possibility is that attention impairments precede, and are a risk factor for developing depression: impaired goal-directed attention could reduce the likelihood of achieving one’s goals, which could lead to lower estimation of one’s capacity to achieve rewards or more frequent failure to avoid punishments, all of which could contribute to the mood-related symptoms of depression. Given that attention abilities vary naturally in the population, it may be the case that those who struggle with attention relative to their peers are at increased risk of developing depression and could benefit from preventative measures to improve attention. Alternatively, it is possible that attention impairments develop in the context of MDD and may have a role in its chronicity. For example, excessive rumination/worry might sap attentional resources, lack of sufficient sleep could produce attention deficits, or extreme anhedonia could lead to general psychomotor slowing. However, it should be noted that these symptoms of depression are often ameliorated with no improvement in attention (^25,100^ see the section “Improving attention”) and our own data suggest that selective attention function does not correlate with insomnia or excessive worrying^20^.

Giollabhui et al.^163^ used a longitudinal sample to investigate selective, sustained, and divided attention, and showed that the interactions among these pathways may be complex. Specifically, baseline-divided attention performance predicted depressive symptoms at follow-up, but higher depressive symptoms at baseline also predicted worse selective attention at follow-up, and childhood stress predicted both higher depressive symptoms and worse attention. Future studies will be essential to probe the relationship between attention and other aspects of depression in greater detail, including the potential role of attention impairments in other clinical profiles associated with poorer prognosis (e.g., anhedonia). Longitudinal studies may also reveal whether attention impairments operate as state or trait-like features of depression, whether attention behaviors co-vary with neurobiological changes over time and how these change with targeted intervention, and whether targeting attention impairments will ultimately improve mood-related symptoms and reduce the burden of MDD.

## Conclusion: getting personal

As the field of precision psychiatry develops, it is becoming increasingly important to understand the nuances of individual variability to develop personalized treatments^164,165^. Cognitive features of psychiatric disorders should not be overlooked in this regard, and more work should be done to determine which individuals would be most likely to benefit from treatments targeting cognitive symptoms like attention impairments. Pinning down precise neurobiological targets using a combination of hypothesis-driven and data-driven approaches will be essential for achieving the overarching goal of developing effective, personalized treatments for attention trans-diagnostically. At present, the best available evidence suggests that symptoms of depression and attention do not generally improve together for most patients with current treatments, particularly SSRIs. Drugs targeting catecholamines (e.g., DA, NE) may benefit sustained attention, but it remains unknown whether these interventions target the specific neural circuit or electrophysiological correlates of goal-directed attentional orienting or whether they simply increase overall arousal levels. Recent innovative approaches to understanding attention behaviorally and biologically^6^ continue to bring us closer to this possible future of personalized psychiatry, but bringing these efforts across the finish line will depend on continued work across disciplines, from our basic understanding of intact attentional systems to our assessments of how these are disrupted in the context of mental illness. Given that attention impairments are a debilitating symptom for many depressed individuals and are not alleviated by current first-line antidepressant treatments, these translational efforts have the potential to dramatically improve individuals’ quality of life.

## Supplementary information

Appendix: Internal vs. External Attention

## References

[1] American Psychiatric Association. (2013). DSM-5 Task Force. Diagnostic and Statistical Manual of Mental Disorders: DSM-5™.

[2] Zuckerman H (2018). Recognition and treatment of cognitive dysfunction in major depressive disorder. Front. Psychiatry.

[3] Fehnel SE (2016). Patient-centered assessment of cognitive symptoms of depression. CNS Spectr..

[4] Cotrena C, Branco LD, Shansis FM, Fonseca RP (2016). Executive function impairments in depression and bipolar disorder: association with functional impairment and quality of life. J. Affect. Disord..

[5] Majer M (2004). Impaired divided attention predicts delayed response and risk to relapse in subjects with depressive disorders. Psychological Med..

[6] Insel TR (2014). The NIMH research domain criteria (RDoC) project: precision medicine for psychiatry. Am. J. Psychiatry.

[7] National Institute of Mental Health. Research Domain Criteria (RDoC): RDoC Constructs: Domain: Cognitive Systems. [Internet]. Retrieved Aug 2019. Available from: https://www.nimh.nih.gov/research/research-funded-by-nimh/rdoc/constructs/cognitive-systems.shtml.

[8] Rock PL, Roiser JP, Riedel WJ, Blackwell AD (2014). Cognitive impairment in depression: a systematic review and meta-analysis. Psychol. Med..

[9] Katsuki F, Constantinidis C (2014). Bottom-up and top-down attention: different processes and overlapping neural systems. Neuroscientist.

[10] Fried EI, Nesse RM (2015). Depression is not a consistent syndrome: an investigation of unique symptom patterns in the STAR*D study. J. Affect. Disord..

[11] James W (1890). The Principles of Psychology..

[12] Ibos G, Freedman DJ (2016). Interaction between spatial and feature attention in posterior parietal cortex. Neuron.

[13] Patzwahl DR, Treue S (2009). Combining spatial and feature-based attention within the receptive field of MT neurons. Vis. Res..

[14] Giesbrecht B, Woldorff MG, Song AW, Mangun GR (2003). Neural mechanisms of top-down control during spatial and feature attention. Neuroimage.

[15] Galashan D, Siemann J (2017). Differences and similarities for spatial and feature-based selective attentional orienting. Front. Neurosci..

[16] Kertzman S (2010). Stroop performance in major depression: selective attention impairment or psychomotor slowness?. J. Affect. Disord..

[17] Holmes AJ, Pizzagalli DA (2008). Response conflict and frontocingulate dysfunction in unmedicated participants with major depression. Neuropsychologia.

[18] Cataldo MG, Nobile M, Lorusso ML, Battaglia M, Molteni M (2005). Impulsivity in depressed children and adolescents: a comparison between behavioral and neuropsychological data. Psychiatry Res..

[19] Degl’Innocenti A, Ågren H, Bäckman L (1998). Executive deficits in major depression. Acta Psychiatr. Scand..

[20] Keller, A. S., Ball, T. M. & Williams, L. M. Deep phenotyping of attention impairments and the “Inattention Biotype” in major depressive disorder. *Psychol. Med.* 1–10 (2019). 10.1017/S0033291719002290. 10.1017/S0033291719002290 PMC8022888 31477195

[21] Ladouceur CD (2012). Altered error-relaed brain activity in youth with major depression. Developmental Cogn. Neurosci..

[22] Olvet DM, Klein DN, Hajcak G (2010). Depression symptom severity and error-related brain activity. Psychiatry Res..

[23] Kemp AH (2009). Fronto-temporal alterations within the first 200 ms during an attentional task distinguish major depression, non-clinical participants with depressed mood, and healthy controls: a potential biomarker?. Hum. Brain Mapp..

[24] Tenke CE (2008). Hemispatial PCA dissociates temporal from parietal ERP generator patterns: CSD components in healthy adults and depressed patients during a dichotic oddball task. Int. J. Psychophysiol..

[25] Gyurak A (2016). Frontoparietal activation during response inhibition predicts remission to antidepressants in patients with major depression. Biol. Psychiatry.

[26] Li X, Wu H, Lou C, Xing B, Yu E (2014). Study on the executive function of attention in depression patients based on SPECT technology. Int J. Clin. Exp. Med.

[27] Li Y (2016). Depression-related brain connectivity analyzed by EEG event-related phase synchrony measure. Front. Human Neurosci..

[28] van Vugt MK, van der Velde M, ESM-MERGE Investigators. (2018). How does rumination impact cognition? A first mechanistic model. Top. Cogn. Sci..

[29] Cherry EC (1953). Some experiments on the recognition of speech, with one and with two ears. J. Acoust. Soc. Am..

[30] McMains SA, Somers DC (2005). Processing efficiency of divided spatial attention mechanisms in human visual cortex. J. Neurosci..

[31] Godefroy O, Rousseaux M (1996). Divided and focused attention in patients with lesion of the prefrontal cortex. Brain Cogn..

[32] Richer F (1993). Target detection deficits in frontal lobectomy. Brain Cogn..

[33] Owen AM, Roberts AC, Polkey CE, Sahakian BJ, Robbins TW (1991). Extra-dimensional versus intra-dimensional set shifting performance following frontal lobe excisions, temporal lobe excisions, or amygdalo-hippocampectomy in man. Neuropsychologia.

[34] Lautenbacher S, Spernal J, Krieg J-C (2002). Divided and selective attention in panic disorder: a comparative study of patients with panic disorder, major depression and healthy controls. Eur. Arch. Psychiatry Clin. Neurosci..

[35] Thomas P, Goudemand M, Rousseaux M (1998). Divided attention in major depression. Psychiatry Res..

[36] Mikoteit T (2015). Improved alertness is associated with early increase in serum brain-derived neurotrophic factor and antidepressant treatment outcome in major depression. Neuropsychobiology.

[37] Kim SJ (2015). The relationship between poor performance on attention tasks and increased suicidal ideation in adolescents. Eur. Child Adolesc. Psychiatry.

[38] LeMoult J, Gotlib IH (2019). Depression: a cognitive perspective. Clin. Psychol. Rev..

[39] Snyder HR (2013). Major depressive disorder is associated with broad impairments on neuropsychological measures of executive function: a meta-analysis and review. Psychological Bull..

[40] National Institute of Mental Health. Research Domain Criteria (RDoC): Cognitive Systems: Workshop Proceedings. [Internet]. Retrieved Aug 2019. Available from: https://www.nimh.nih.gov/research/research-funded-by-nimh/rdoc/cognitive-systems-workshop-proceedings.shtml (2019).

[41] Chun MM, Golomb JD, Turk-Browne NB (2011). A taxonomy of external and internal attention. Annu Rev. Psychol..

[42] Gratton G, Cooper P, Fabiani M, Carter CS, Karayanidis F (2018). Dynamics of cognitive control: theoretical bases, paradigms, and a view for the future. Psychophysiology.

[43] Carrasco M, Barbot A (2019). Spatial attention alters visual appearance. Curr. Opin. Psychol..

[44] Liu T, Abrams J, Carrasco M (2009). Voluntary attention enhances contrast appearance. Psychol. Sci..

[45] Fuller S, Carrasco M (2006). Exogenous attention and color perception: performance and appearance of saturation and hue. Vis. Res..

[46] Störmer VS, Alvarez GA (2016). Attention alters perceived attractiveness. Psychological Sci..

[47] Mishra MV, Srinivasan N (2017). Exogenous attention intensifies perceived emotion expressions. Neurosci. Conscious.

[48] Bubl E, Kern E, Ebert D, Bach M, van Elst LT (2010). Seeing gray when feeling blue? Depression can be measured in the eye of the diseased. Biol. Psychiatry.

[49] Barbot A, Carrasco M (2018). Emotion and anxiety potentiate the way attention alters visual appearance. Sci. Rep..

[50] Krajbich I (2019). Accounting for attention in sequential sampling models of decision making. Curr. Opin. Psychol..

[51] Shimojo S, Simon C, Shimojo E, Scheier C (2003). Gaze bias both reflects and influences preference. Nat. Neurosci..

[52] Chelazzi L (2014). Altering spatial priority maps via reward-based learning. J. Neurosci..

[53] Niv Y (2015). Reinforcement learning in multidimensional environments relies on attention mechanisms. J. Neurosci..

[54] Leong Y-C, Radulescu A, Daniel R, DeWoskin V, Niv Y (2017). Dynamic interaction between reinforcement learning and attention in multidimensional environments. Neuron.

[55] Huys QJM, Daw ND, Dayan P (2015). Depression: a decision-theoretic analysis. Annu. Rev. Neurosci..

[56] Macleod C, Mathews A, Tata P (1986). Attentional bias in emotional disorders. J. Abnorm. Psychol..

[57] Williams JM, Mathews A, MacLeod C (1996). The emotional stroop task and psychopathology. Psychol. Bull..

[58] Gotlib IH, Joormann J (2010). Cognition and depression: current status and future directions. Annu Rev. Clin. Psychol..

[59] Kircanski K, Gotlib IH (2015). Processing of emotional information in major depressive disorder: toward a dimensional understanding. Emot. Rev..

[60] Lang PJ, Davis M (2006). (2006). Emotion, motivation, and the brain: reflex foundations in animal and human research. Prog. Brain Res..

[61] Clasen PC, Wells TT, Ellis AJ, Beevers CG (2013). Attentional biases and the persistence of sad mood in major depressive disorder. J. Abnorm Psychol..

[62] Vanlessen N, De Raedt R, Koster EHW, Pourtois G (2016). Happy heart, smiling eyes: a systematic review of positive mood effects on broadening of visuospatial attention. Neurosci. Biobehav. Rev..

[63] Meeten F, Davey GCL (2011). Mood-as-input hypothesis and perseverative psychopathologies. Clin. Psychol. Rev..

[64] Fredrickson B (2001). The role of positive emotions in positive psychology: the broaden-and-build theory of positive emotions. Am. Psychol..

[65] Isen AM, Daubman KA, Nowicki GP (1987). Positive affect facilitates creative problem-solving. J. Pers. Soc. Psychol..

[66] Isen AM, Daubman KA (1984). The influence of affect on categorization. J. Personal. Soc. Psychol..

[67] Derryberry, D, Tucker, D. M, Neidenthal, P. M. & Kitayama, S. Motivating the focus of attention. In *The Heart’s Eye: Emotional Influences in Perception and Attention*. (eds Niedenthal, P. M. & Kitayama, S.) 167–196 (Academic: San Diego, CA, 1994).

[68] Easterbrook J (1959). The effect of emotion on cue utilization and the organization of behavior. Psychological Rev..

[69] Loftus E (1979). Eyewitness Testimony.

[70] Brand N, Verspui L, Oving A (1997). Induced mood and selective attention. Percept. Mot. Skills.

[71] Storbeck J, Clore GL (2008). Affective arousal as information: how affective arousal influences judgments, learning, and memory. Soc. Personal. Psychol. Compass.

[72] Kastner S, Pinsk MA, De Weerd P, Desimone R, Ungerleider LG (1999). Increased activity in human visual cortex during directed attention in the absence of visual stimulation. Neuron.

[73] Corbetta M (1998). Frontoparietal cortical networks for directing attention and the eye to visual locations: identical, independent, or overlapping neural systems?. PNAS.

[74] Nobre AC (1997). Functional localization of the system for visuospatial attention using positron emission tomography. Brain.

[75] Corbetta M, Shulman GL (2002). Control of goal-directed and stimulus-driven attention in the brain. Nat. Rev. Neurosci..

[76] Falkenberg LE, Specht K, Westerhausen R (2011). Attention and cognitive control networks assessed in a dichotic listening fmri study. Brain Cogn..

[77] Bassett DS (2011). Dynamic reconfiguration of human brain networks during learning. PNAS.

[78] Gu S (2015). Controllability of structural brain networks. Nat. Commun..

[79] Gu S (2017). Optimal trajectories of brain state transitions. NeuroImage.

[80] Qin J (2014). Abnormal brain anatomical topological organization of the cognitive-emotional and the frontoparietal circuitry in major depressive disorder. Magn. Reson. Med..

[81] Luo Q (2015). Frequency dependent topological alterations of intrinsic functional connectome in major depressive disorder. Sci. Rep..

[82] He Y (2018). Reconfiguration of cortical networks in MDD uncovered by multiscale community detection with fMRI. Cereb. Cortex.

[83] Gratton C, Sun H, Petersen SE (2018). Control networks and hubs. Psychophysiology.

[84] Dixon ML (2018). Heterogeneity within the frontoparietal control network and its relationship to the default and dorsal attention networks. PNAS.

[85] Ahrens M-M, Veniero D, Freund IM, Harvey M, Thut G (2019). Both dorsal and ventral attention network nodes are implicated in exogenously driven visuospatial anticipation. Cortex.

[86] Liu Y (2017). Deciding where to attend: large-scale network mechanisms underlying attention and intention revealed by graph-theoretic analysis. NeuroImage.

[87] Fiebelkorn IC, Pinsk MA, Kastner S (2018). A dynamic interplay within the frontoparietal network underlies rhythmic spatial attention. Neuron.

[88] Michalka SW, Kong L, Rosen ML, Shinn-Cunningham BG, Somers DC (2015). Short-term memory for space and time flexibly recruit complementary sensory-biased frontal lobe attention networks. Neuron.

[89] Noyce AL, Cestero N, Michalka SW, Shinn-Cunningham BG, Somers DC (2017). Sensory-biased and multiple-demand processing in human lateral frontal cortex. J. Neurosci..

[90] Pfurtscheller G, Stancák A, Neuper C (1996). Event-related synchronization (ERS) in the alpha band–an electrophysiological correlate of cortical idling: a review. Int J. Psychophysiol..

[91] Payne L, Sekuler R (2014). On the importance of ignoring: alpha oscillations protect selective processing. Curr. Directions Psychological Sci..

[92] Mazaheri A (2014). Region-specific modulations in oscillatory alpha activity serve to facilitate processing in the visual and auditory modalities. Neuroimage.

[93] Kelly SP, Lalor EC, Reilly RB, Foxe JJ (2006). Increases in alpha oscillatory power reflect an active retinotopic mechanism for distracter suppression during sustained visuospatial attention. J. Neurophysiol..

[94] Romei V, Rihs T, Brodbeck V, Thut G (2008). Resting electroencephalogram alpha- power over posterior sites indexes baseline visual cortex excitability. NeuroReport.

[95] Cavanagh JF, Frank MJ (2014). Frontal theta as a mechanism for cognitive control. Trends Cogn. Sci..

[96] Cavanagh JF, Zambrano-Vazquez L, Allen JJB (2011). Theta lingua franca: a common mid-frontal substrate for action monitoring processes. Psychophysiology.

[97] Keller AS, Payne L, Sekuler R (2017). Characterizing the roles of alpha and theta oscillations in multisensory attention. Neuropsychologia.

[98] Fellrath J, Mottaz A, Schnider A, Guggisberg AG, Ptak R (2016). Theta-band functional connectivity in the dorsal fronto-parietal network predicts goal-directed attention. Neuropsychologia.

[99] Shilyansky C (2016). Effect of antidepressant treatment on cognitive impairments associated with depression: a randomised longitudinal study. Lancet Psychiatry.

[100] Luo LL (2013). A distinct pattern of memory and attention deficiency in patients with depression. Chin. Med. J..

[101] Reppermund S (2007). Persistent cognitive impairment in depression: the role of psychopathology and altered hypothalamic-pituitary-adrenocortical (HPA) system regulation. Biol. Psychiatry.

[102] Knorr U, Madsen JM, Kessing LV (2019). The effect of selective serotonin reuptake inhibitors in healthy subjects revisited: a systematic review of the literature. Exp. Clin. Psychopharmacol..

[103] Mahableshwarkar AR, Zajecka J, Jacobson W, Chen Y, Keefe RS (2015). A randomized, placebo-controlled, active-reference, double-blind, flexible-dose study of the efficacy of vortioxetine on cognitive function in major depressive disorder. Neuropsychopharmacology.

[104] McIntyre RS (2017). Efficacy of vortioxetine on cognitive functioning in working patients with major depressive disorder. J. Clin. psychiatry.

[105] Arnsten AF (2011). Catecholamine influences on dorsolateral prefrontal cortical networks. Biol. Psychiatry.

[106] Corp SA, Gitlin MJ, Altshuler LL (2014). A review of the use of stimulants and stimulant alternatives in treating bipolar depression and major depressive disorder. J. Clin. Psychiatry.

[107] Tamminga HG, Reneman L, Huizenga HM, Geurts HM (2016). Effects of methylphenidate on executive functioning in attention-deficit/hyperactivity disorder across the lifespan: a meta-regression analysis. Psychol. Med..

[108] Linssen AM, Sambeth A, Vuurman EF, Riedel WJ (2014). Cognitive effects of methylphenidate in healthy volunteers: a review of single dose studies. Int J. Neuropsychopharmacol..

[109] Rapoport JL (1980). Dextroamphetamine. Its cognitive and behavioral effects in normal and hyperactive boys and normal men. Arch. Gen. Psychiatry.

[110] MacQueen DA (2018). Amphetamine improves mouse and human attention in the 5-choice continuous performance test. Neuropharmacology.

[111] Paton K (2014). Methylphenidate improves some but not all measures of attention, as measured by the TEA-Ch in medication-naive children with ADHD. Child Neuropsychol..

[112] ter Huurne N (2015). Methylphenidate alters selective attention by amplifying salience. Psychopharmacol. (Berl.).

[113] Servan-Schreiber D, Carter CS, Bruno RM, Cohen JD (1998). Dopamine and the mechanisms of cognition: Part II. D-amphetamine effects in human subjects performing a selective attention task. Biol. Psychiatry.

[114] Conners CK (1996). Bupropion hydrochloride in attention deficit disorder with hyperactivity. J. Am. Acad. Child Adolesc. Psychiatry.

[115] Acheson A, de Wit H (2008). Bupropion improves attention but does not affect impulsive behavior in healthy young adults. Exp. Clin. Psychopharmacol..

[116] Cope ZA (2017). Modafinil improves attentional performance in healthy, non-sleep deprived humans at doses not inducing hyperarousal across species. Neuropharmacology.

[117] Ikeda Y (2017). Modafinil enhances alerting-related brain activity in attention networks. Psychopharmacol. (Berl.).

[118] Turner DC, Clark L, Dowson J, Robbins TW, Sahakian BJ (2004). Modafinil improves cognition and response inhibition in adult attention-deficit/hyperactivity disorder. Biol. Psychiatry.

[119] DeBattista C, Lembke A, Solvason HB, Ghebremichael R, Poirier J (2004). A prospective trial of modafinil as an adjunctive treatment of major depression. J. Clin. Psychopharmacol..

[120] Jung KY (2015). Sternberg working memory performance following treatment with pramipexole in patients with moderate-to-severe restless legs syndrome. Sleep. Med..

[121] Faraone SV (2005). Atomoxetine and stroop task performance in adult attention-deficit/hyperactivity disorder. J. Child Adolesc. Psychopharmacol..

[122] Tian Y (2016). Venlafaxine treatment reduces the deficit of executive control of attention in patients with major depressive disorder. Sci. Rep..

[123] Gualtieri CT, Johnson LG (2007). Bupropion normalizes cognitive performance in patients with depression. MedGenMed.

[124] Siepmann T (2008). The effects of venlafaxine on cognitive functions and quantitative EEG in healthy volunteers. Pharmacopsychiatry.

[125] Greer TL, Dunderajan P, Grannemann BD, Kurian BT, Trivedi MH (2014). Does duloxetine improve cognitive function independently of its antidepressant effect in patients with major depressive disorder and subjective reports of cognitive dysfunction?. Depress Res. Treat..

[126] Taylor FB, Russo J (2001). Comparing guanfacine and dextroamphetamine for the treatment of adult attention-deficit/hyperactivity disorder. J. Clin. Psychopharmacol..

[127] Arnsten AF (2015). Stress weakens prefrontal networks: molecular insults to higher cognition. Nat. Neurosci..

[128] Berridge CW, Arnsten AF (2013). Psychostimulants and motivated behavior: arousal and cognition. Neurosci. Biobehav Rev..

[129] van Belkum SM, Bosker FJ, Kortekaas R, Beersma DG, Schoevers RA (2016). Treatment of depression with low-strength transcranial pulsed electromagnetic fields: a mechanistic point of view. Prog. Neuropsychopharmacol. Biol. Psychiatry.

[130] Marek S, Dosenbach NUF (2018). The frontoparietal network: function, electrophysiology, and importance of individual precision mapping. Dialogues Clin. Neurosci..

[131] Hwang JH, Kim SH, Park CS, Bang SA, Kim SE (2010). Acute high-frequency rTMS of the left dorsolateral prefrontal cortex and attentional control in healthy young men. Brain Res..

[132] Levkovitz Y (2009). Deep transcranial magnetic stimulation over the prefrontal cortex: evaluation of antidepressant and cognitive effects in depressive patients. Brain Stimul..

[133] Naim-Feil J (2016). Neuromodulation of attentional control in major depression: a Pilot DeepTMS study. Neural plasticity.

[134] Vanderhasselt MA, De Raedt R, Baeken C, Leyman L, D’haenen H (2009). A single session of rTMS over the left dorsolateral prefrontal cortex influences attentional control in depressed patients. World J. Biol. Psychiatry.

[135] Loo CK (2012). Transcranial direct current stimulation for depression: 3-week, randomised, sham-controlled trial. Br. J. Psychiatry.

[136] Iimori T (2019). Effectiveness of the prefrontal repetitive transcranial magnetic stimulation on cognitive profiles in depression, schizophrenia, and Alzheimer’s disease: a systematic review. Prog. Neuropsychopharmacol. Biol. Psychiatry.

[137] Martin DM, McClintock SM, Forster J, Loo CK (2016). Does therapeutic repetitive transcranial magnetic stimulation cause cognitive enhancing effects in patients with neuropsychiatric conditions? A systematic review and meta-analysis of randomized controlled trials. Neuropsychol. Rev..

[138] Demirtas-Tatlidede A, Vhabzadeh-Hagh AM, Pascual-Leone A (2012). Can non-invasive brain stimulation enhance cognition in neuropsychiatric disorders?. Neuropharmacology.

[139] Deslandes AC (2010). Effect of aerobic training on EEG alpha asymmetry and depressive symptoms in the elderly: a 1-year follow-up study. Braz. J. Med. Biol. Res..

[140] Smith PJ (2010). Aerobic exercise and neurocognitive performance: a meta-analytic review of randomized controlled trials. Psychosom. Med..

[141] Kubesch S (2003). Aerobic endurance exercise improves executive functions in depressed patients. J. Clin. Psychiatry.

[142] Vasques PE, Moraes H, Silveira H, Deslandes AC, Laks J (2011). Acute exercise improves cognition in the depressed elderly: the effect of dual-tasks. Clinics.

[143] Greer TL, Furman JL, Trivedi MH (2017). Evaluation of the benefits of exercise on cognition in major depressive disorder. Gen. Hospital Psychiatry.

[144] Brondino N (2017). A systematic review of cognitive effects of exercise in depression. Acta Psychiatr. Scandinavica.

[145] Baer RA (2003). Mindfulness training as a clinical intervention: a conceptual and empirical review. Clin. Psychol.: Sci. Pract..

[146] Chiesa A, Calati R, Serretti A (2011). Does mindfulness training improve cognitive abilities? A systematic review of neuropsychological findings. Clin. Psychol. Rev..

[147] Wielgosz J, Goldberg SB, Kral TRA, Dunne JD, Davidson RJ (2019). Mindfulness meditation and psychopathology. Annu Rev. Clin. Psychol..

[148] Goldberg SB (2019). Mindfulness-based cognitive therapy for the treatment of current depressive symptoms: a meta-analysis. Cogn. Behav. Ther..

[149] Kerr CE (2011). Effects of mindfulness meditation training on anticipatory alpha modulation in primary somatosensory cortex. Brain Res. Bull..

[150] Bhayee S (2016). Attentional and affective consequences of technology supported mindfulness training: a randomised, active control, efficacy trial. BMC Psychol..

[151] Anguera JA (2013). Video game training enhances cognitive control in older adults. Nature.

[152] Anguera JA, Gunning FM, Areán PA (2017). Improving late life depression and cognitive control through the use of therapeutic video game technology: A proof‐of‐concept randomized trial. Depression Anxiety.

[153] Motter JN (2016). Computerized cognitive training and functional recovery in major depressive disorder: a meta-analysis. J. Affect. Disord..

[154] Arean PA (2016). The use and effectiveness of mobile apps for depression: results from a fully remote clinical trial. J. Med. Internet Res..

[155] Iwata M, Ota KT, Duman RS (2013). The inflammasome: pathways linking psychological stress, depression, and systemic illnesses. Brain, Behav., Immun..

[156] Burke HM, Davis MC, Otte C, Mohr DC (2005). Depression and cortisol responses to psychological stress: a meta-analysis. Psychoneuroendocrinology.

[157] Sapolsky RM (2000). Glucocorticoids and hippocampal atrophy in neuropsychiatric disorders. Arch. Gen. Psychiatry.

[158] Wellman CL (2001). Dendritic reorganization in pyramidal neurons in medial prefrontal cortex after chronic corticosterone administration. J. Neurobiol..

[159] Hyman JM, Zilli EA, Paley AM, Hasselmo ME (2010). Working memory performance correlates with prefrontal-hippocampal theta interactions but not with prefrontal neuron firing rates. Front. Integr. Neurosci..

[160] Yu JY, Frank LM (2015). Hippocampal-cortical interaction in decision making. Neurobiol. Learn. Mem..

[161] Belleau EL, Treadway MT, Pizzagalli DA (2019). The impact of stress and major depressive disorder on hippocampal and medial prefrontal cortex morphology. Biol. Psychiatry.

[162] Vertes RP (2006). Interactions among the medial prefrontal cortex, hippocampus and midline thalamus in emotional and cognitive processing in the rat. Neuroscience.

[163] Giollabhui NM, Olino TM, Nielsen J, Abramson LY, Alloy LB (2019). Is worse attention a risk factor for or a consequence of depression, or are worse attention and depression better accounted for by stress? A prospective test of three hypotheses. Clin. Psychological Sci..

[164] Fernandes BS (2017). The new field of ‘precision psychiatry’. BMC Med..

[165] Williams LM (2016). Precision psychiatry: a neural circuit taxonomy for depression and anxiety. Lancet Psychiatry.

