# Correction: Paying attention to attention in depression

**DOI:** 10.1038/s41398-020-0748-3

**Published:** 2020-02-12

**Authors:** Arielle S. Keller, John E. Leikauf, Bailey Holt-Gosselin, Brooke R. Staveland, Leanne M. Williams

**Affiliations:** 1 Graduate Program in Neurosciences, Stanford University, Stanford, CA USA; 2 Department of Psychiatry and Behavioral Sciences, Stanford University, Stanford, CA USA

**Keywords:** Depression, Human behaviour

**Correction to: Translational Psychiatry**

10.1038/s41398-019-0616-1 published online 7 November 2019.

In the original Article, Naoise Mac Giollabhui was incorrectly cited as “Giollabhui, N. M” instead of “Mac Giollabhui, N” in reference 163. This has been updated in the HTML and PDF versions of this Article.

