# Supplementary material for: Paying attention to attention in depression
Source: Transl Psychiatry. 2019 Nov 7;9:279. doi: 10.1038/s41398-019-0616-1 (PMC6838308; doi:10.1038/s41398-019-0616-1)
Supplement: Supplementary file 1 — Appendix: Internal vs. External Attention [file 41398_2019_616_MOESM1_ESM.docx]

**Internal vs. External Attention**

Prior studies investigating changes in “internal” attention have reported increased self-focused attention (related to self-referential processing) in depressed individuals (1) and with negative affect generally (2). Broadly speaking, internal and external attention are defined by whether the focus of attention is on internal representations (e.g., visual working memory) or external sensory stimuli (3), such that instances of either internal or external attention could also be defined as selective, sustained, or divided attention. Neurobiological studies probing internally vs. externally-focused attention have primarily found overlap in the fronto-parietal systems (discussed in detail in Section “Putative Mechanisms of Attention Impairment: Neural Circuits”) used to guide attention toward internal representations and external stimuli, though smaller-scale differences between the two emerge as well (e.g. differing multi-voxel patterns in the superior parietal lobule (4) and a shift in the balance between frontal and parietal recruitment (5-7).

It is plausible that dysfunction within large-scale fronto-parietal goal-directed attention systems may impair the orienting of attention toward both internal and external stimuli in selective, sustained, and divided attention conditions. In this model, negatively-valenced internal or external stimuli may capture attention initially, and lingering attention on this negative information may arise from an inability to re-orient attention (either internally or externally) in a goal-directed manner. This model fits with empirical data demonstrating that depressed and dysphoric individuals exhibit deficits in internal switching of attention with emotional material (8, 9), though a causal link between fronto-parietal dysfunction and changes in internally-guided attention (e.g., self-reflection, rumination) in depressed individuals has not been demonstrated. One possibility is that deficits in internal attention impede one’s ability to evaluate one’s own cognitive experiences at a meta-cognitive level, which could provide an explanation for the lack of correspondence between clinician/self-reported inattention and attention impairments measured behaviorally (10).

**Supplemental References**

1. Ingram RE, Smith TW. Depression and internal versus external focus of attention. *Cognitive Therapy and Research* 1984; **8**(2):139-151.
2. Mor N, Winquist J. Self-focused attention and negative affect: a meta-analysis. *Psychol Bull.* 2002; **128**(4):638-662.
3. Chun MM, Golomb JD, Turk-Browne NB. A taxonomy of external and internal attention. *Annu Rev Psychol* 2011; **62**:73-101.
4. Esterman M, Chiu Y-C, Tamber-Rosenau BJ, Yantis S. Decoding cognitive control in human parietal cortex. *PNAS* 2009; **106**(42):17974-17979.
5. Tanoue RT, Jones KT, Peterson DJ, Berryhill ME. Differential frontal involvement in shifts of internal and perceptual attention. *Brain Stimul.* 2013; **6**(4):675-682.
6. Lepsien J, Nobre AC. Cognitive control of attention in the human brain: insights from orienting attention to mental representations. *Brain Res* 2006; **1105**(1):20-31.
7. Nobre AC et al.; Orienting attention to locations in perceptual versus mental representations. *J Cogn Neurosci* 2004; **16**(3):363-373.
8. Wante L, Mueller SC, Demeyer I, Naets T, Braet C. Internal shifting impairments in response to emotional information in dysphoric adolescents. *J Behav Ther Exp Psychiatry* 2017; **57**:70-79.
9. Lo BC, Allen NB. Affective bias in internal attention shifting among depressed youth. *Psychiatry Res* 2011; **187**(1-2):125-129.
10. Keller AS, Ball TM, Williams LM. Deep phenotyping of attention impairments and the “Inattention Biotype” in major depressive disorder. *Psychological Medicine* 2019; in press*.*
